# Neighborhood walkability, physical activity, and walking for transportation: A cross-sectional study of older adults living on low income

**DOI:** 10.1186/s12877-017-0469-5

**Published:** 2017-04-10

**Authors:** Anna M. Chudyk, Heather A. McKay, Meghan Winters, Joanie Sims-Gould, Maureen C. Ashe

**Affiliations:** 1 Centre for Hip Health and Mobility, 7th floor—2635 Laurel Street, Vancouver, BC V5Z 1M9 Canada; 2 Department of Family Practice, University of British Columbia, 3rd Floor - 5950 University Boulevard, Vancouver, BC V6T 1Z3 Canada; 3 Faculty of Health Sciences, Simon Fraser University, 11522 - 8888 University Drive, Burnaby, BC V5A 1S6 Canada

**Keywords:** Built environment, Walkability, Walk Score, Physical activity, Walking, Walking for transportation

## Abstract

**Background:**

Walking, and in particular, outdoor walking, is the most common form of physical activity for older adults. To date, no study investigated the association between the neighborhood built environment and physical activity habits of older adults of low SES. Thus, our overarching aim was to examine the association between the neighborhood built environment and the spectrum of physical activity and walking for transportation in older adults of low socioeconomic status.

**Methods:**

Cross-sectional data were from the Walk the Talk Study, collected in 2012. Participants (*n* = 161, mean age = 74 years) were in receipt of a rental subsidy for low income individuals and resided in neighbourhoods across Metro Vancouver, Canada. We used the Street Smart Walk Score to objectively characterize the built environment main effect (walkability), accelerometry for objective physical activity, and the Community Healthy Activities Model Program for Seniors (CHAMPS) questionnaire to measure walking for transportation. We used regression analyses to examine associations of objectively measured physical activity [total volume, light intensity and moderate intensity physical activity (MVPA)] and self-reported walking for transportation (any, frequency, duration) with walkability. We adjusted analyses for person- and environment-level factors associated with older adult physical activity.

**Results:**

Neighbourhood walkability was not associated with physical activity volume or intensity and self-reported walking for transportation, with one exception. Each 10-point increase in Street Smart Walk Score was associated with a 45% greater odds of any walking for transportation (compared with none; OR = 1.45, 95% confidence interval = 1.18, 1.78). Sociodemographic, physical function and attitudinal factors were significant predictors of physical activity across our models.

**Conclusions:**

The lack of associations between most of the explored outcomes may be due to the complexity of the relation between the person and environment. Given that this is the first study to explore these associations specifically in older adults living on low income, this study should be replicated in other settings.

## Background

Despite the many benefits of a physically active lifestyle [1], adults aged ≥ 60 years represent the least active age group [2, 3]; only 13% of older adults in Canada [2] attain sufficient physical activity to meet public health guidelines of engaging in ≥ 150 min of moderate-to-vigorous physical activity (MVPA) per week [4, 5]. Barriers to engaging in physical activity in older adults include poor health, unsupportive built environments (e.g., no sidewalks, parks or recreation centres), lack of knowledge about the relationship between physical activity and health and negative experiences with exercise earlier in life [6]. Further, given the broad spectrum of mobility-disability in the older adult population, achieving guideline levels of higher (moderate) intensity physical activity may not be possible. Although often overlooked, physical activity at levels below guidelines is important to the general health, mobility and community engagement of older people. For example, total physical activity volume may have stronger associations with cardiometabolic biomarkers than MVPA accumulated in bouts [7]. Light-intensity physical activity was associated with older adults’ physical health and well-being, independent of MVPA [8]. It is also plausible that individuals who are more physically active outdoors have more opportunities for community engagement. Thus, although current physical activity guidelines are important for health, encouraging any physical activity, including light physical activity, is increasingly recognized as important [9, 10]. Moreover, given the broad range of mobility limitations for some older adults, this may be more a more realistic public health goal for older adults.

Walking, and in particular, outdoor walking, is the most common form of physical activity for older adults [11]. Walking requires minimal equipment, and intensity is dictated by the individual. Further, within a supportive outdoor environment, walking can be incorporated relatively easily into daily life routines as either structured or incidental activity. The built environment, defined as urban design, land use, and transportation systems [12], can be an important facilitator or a barrier to outdoor walking. For example, a built environment rich with destinations relevant to older adults provides an opportunity to walk for daily travel [13–16]. Yet findings are mixed regarding specific built environment features associated with older adult walking, and physical activity in general [17–20]. Built environment features most consistently associated with older adult walking and physical activity include street connectivity, access to destinations (e.g., shops, restaurants) and features related to perceived safety (e.g., good lighting, absence of crime, presence of crosswalks) [18, 20]. The extent to which an individual successfully navigates his/her environment is a result of the match between the pressures exerted by the environment (e.g., features of the built environment) and the competence (e.g., capacity) of the individual [21]. Person-level factors that contribute to older adults’ capacity to be active in their neighbourhood include cognitive, physical, psychosocial, and financial domains; the social environment also plays an important role [22, 23]. Importantly, older adults span diverse physical, psychosocial and cognitive abilities and person-level factors play a key role in the person-environment interaction. Thus, it is relevant to focus on distinct subgroups within the older adult population [e.g., those with mobility limitations or of low socioeconomic status (SES)] to better understand the association between the built environment and older adult physical activity.

Older adults of low SES are understudied in physical activity and aging research [24]. The built environment may more strongly influence the physical activity habits of this population specifically, as they have less disposable income and as a result may rely more upon unstructured (and free) physical activities, such as outdoor walking. Further, older adults of low SES are more likely to walk or take public transit, instead of drive, as their main form of transportation [13, 25]. In doing so they may accrue incidental physical activity, as well as engage with the built environment and other people. Conversely, individuals of low SES are at increased risk of poor health outcomes (e.g., morbidity, physical impairment) that decrease their capacity to be active [26–28]. In sum, older adults of low SES may be more likely to be active in the built environment and may also have health concerns that require their physical activities take place in walkable environments.

Our overarching aim was to examine the association between the neighbourhood built environment and physical activity of older adults living on low income across a spectrum of physical activity.

## Methods

### Aims

Our primary aim was to study the association between the built environment and the total physical activity volume (as measured by accelerometry) of older adults living on low income, including: i) total activity counts (TAC) and ii) steps. Our secondary aims were to determine the association between the built environment and specific intensities and domains of physical activity including: i) light physical activity, ii) MVPA, and iii) self-reported walking for transportation.

### Design and study sample

We conducted a cross-sectional study of older adults who were participants in Walk the Talk, a larger study that investigated the association between the built environment and mobility and health of older adults living on low income. We provide a detailed description of Walk the Talk study methods, including recruitment, outcomes and data collection, elsewhere [15]. Briefly, we identified older adults in receipt of a rental subsidy (Shelter Aid for Elderly Renters, SAFER) through a provincial crown organization (BC Housing). In January—February 2012, we recruited older adults (aged ≥ 65 years) using a stratified design, randomly selecting households (n_total_ = 2000) in their study area (Metro Vancouver) across strata (deciles) of Walk Score® (www.walkscore.com). Upper cut-points (deciles) were 100(1), 93(2), 87(3), 78(4), 72(5), 67(6), 60(7), 52(8), 43(9), and 32(10). Walk Score is a publicly available index that measures the walkability of a street address based on its distance to pre-defined destination categories (e.g., grocery stores, etc.). We excluded individuals who: self-reported a medical diagnosis of dementia, did not understand or speak English, stated that they left their home to go into their community less than once in a typical week, stated that they were unable to walk ≥ 10-m with or without a mobility aid (e.g., cane, walker), and/or were unable to participate in a mobility assessment that involved a 4-m walk. A total of 161 older adults volunteered to be measured in March-May 2012.

### Measures and instruments

#### Outcome measures

##### *Physical activity*

We used ActiGraph GT3X+ (LLC, Pensacola, FL) tri-axial accelerometers to objectively assess participants’ patterns of physical activity. During the in-person measurement sessions, we instructed participants on accelerometer use, including proper placement (e.g., just above right hip and in line with the middle of the right hip, underneath or on top of clothing as long as it fit snugly on the body), wear period (during waking hours, for seven consecutive days), and to remove the accelerometer during water-based activity. We also provided participants with paper copies of these instructions (with photos) to take home. We requested that participants wear their accelerometers on their right hip during waking hours, in the week following their in-person assessment. We collected data continuously (at 30 Hz) and then reintegrated the data to 60-s epochs; we considered more than 60 min of continuous zeroes as non-wear time. To be as inclusive as possible, we chose an 8-h/day wear time criteria for accelerometry data [29]. As this decision had the potential to influence our findings, we conducted sensitivity analyses and found that estimates of our main effect remained stable when we used a more conservative 10-h valid day wear time criterion (data not shown). We excluded from our analyses participants with less than three valid wear days. We used cut-points proposed by Freedson and colleagues to classify time spent in light physical activity (100–1951 counts per minute) and MVPA ( ≥ 1952 counts per minute) [30]. We measured participants’ total volume of physical activity per day using TAC [31] and steps. We calculated TAC (n/day), steps (n/day), light physical activity (min/day), and MVPA (min/day) as total amount of activity accumulated during valid days divided by number of valid days. We processed accelerometry data using ActiLife software version 6.5.4 (LLC, Pensacola, FL).

##### *Self-reported walking for transportation*

We assessed self-reported walking for transportation [yes/no; frequency (n_trips_/wk) and duration (hr/wk)] using a single item from the Community Healthy Activities Model Program for Seniors (CHAMPS) survey [32]. The item asked participants whether in a typical week in the last 4 weeks they had walked to do errands such as going to/from a store or taking children to school (walked for transportation). Participants that reported having walked for transportation were also asked to indicate the frequency (n_trips_/wk) and duration (hr/wk) spent walking for transportation. Response options for the duration component of the question were < 1 h, 1–2.5 h, 3–4.5 h, 5–6.5 h, 7–8.5 h and ≥ 9 h. We used the midpoint of each response option and recoded values to derive duration of walking for transportation (hr/wk); a value of 9.75 hr/wk represented the highest possible duration of walking for transportation [32].

#### Independent variables

We organize independent variables by domains adapted from Webber and colleague’s framework of older adult mobility [23]. This includes a neighbourhood social environment domain to control for (i) neighbourhood social cohesion (e.g., shared beliefs and expectations) and (ii) neighbourhood physical and social disorder that influence physical activity [22].

##### Built environment domain

We used the Street Smart Walk Score® as an objective measure of walkability for participants’ neighbourhood built environment. This was our main effect of interest. The Street Smart Walk Score is a revised version of the Walk Score that uses an updated algorithm to measure the walkability of an address. The algorithm assigns an address a score of 0 to 100 based on network distances from the address to nine different amenity (destination) categories (e.g., grocery stores, restaurants, shopping). Different weights are assigned to different categories based on importance to walkability. Multiple destinations within each category count toward the score in order to reflect depth of choice. Destinations located within ≤ 0.25 miles are assigned maximum scores and those located > 1.5 miles are not factored into the score. The score is penalized (maximum penalty of 10% of total score) for street network characteristics (intersection density and block length) that do not support pedestrian friendliness. The validity of Street Smart Walk Score was established in community-dwelling older adults who reside in the USA [33], in Canadian communities that span a rural-urban continuum [34], and using common measures of the built environment across different buffer sizes [35].

We assessed participants’ perceptions of neighbourhood aesthetics and safety (traffic, crime) using a modified version of the Neighbourhood Environment Walkability Scale—abbreviated (NEWS-A) [36]. Scale scores range from 1 to 4 (strongly disagree, somewhat disagree, somewhat agree, strongly agree). We recoded some items so that higher scores signify higher walkability for all three NEWS-A subscales.

##### Neighbourhood social environment domain

We measured participants’ perceptions of neighbourhood social cohesion and trust using a five-item measure (scale range 1–5) [37]. We used a five-item measure drawn from the Project on Human Development in Chicago Neighbourhoods to measure participants’ perceptions of neighbourhood physical and social disorder (scale range 1–4) [38]. Since we adapted this measure, we provide details about the questions for reproducibility. The items address: how much i) broken glass or trash participants see on neighbourhood sidewalks and streets, and ii) graffiti participants see on neighbourhood buildings and walls, iii) how many vacant/deserted houses or storefront participants see in their neighbourhood; how often iv) participants see people drinking in public places in their neighbourhood, and v) participants see unsupervised children hanging out on the street in their neighbourhood. We reverse coded items. Thus, higher scores indicate more positive perceptions (less disorder).

##### *Physical domain*

We used a TANITA Electronic Scale Model BWB-800 and Seca Stadiometer Model 242 to measure participants’ weight (kg) and height (cm), respectively; we used these data to calculate body mass index (BMI; kg/m^2^). We used the Functional Comorbidity Index to measure self-reported number of comorbidities associated with physical function (scale range 0–18) [39]. Finally, we calculated participants’ gait speed (m/s) as part of the 4-m walk (usual pace) component of the Short Physical Performance Battery [40].

##### *Psychosocial domain*

We measured how much participants like to walk outside using a five-point scale (not at all, not much, neutral, somewhat, very much). We dichotomized (very much vs. other) responses as a majority of responses were in the “very much” category. We used the Ambulatory Self-Confidence Questionnaire to measure participants’ perceived self-efficacy to walk in 22 different home and community environments (scale range 1–10) [41].

##### *Sociodemographic factors*

We used a self-report questionnaire to determine participants’ age, gender, marital status, living arrangement, vehicle access in the last 7 days (yes/no), and dog ownership (yes/no).

#### Analysis

We summarized continuous data using means and standard deviations (SD) and categorical data using counts and percentages. We present summaries by gender, as it is a well-established determinant of older adult physical activity [42].

We fitted multivariable models (linear regression, logistic regression, Poisson regression, described in detail below) as per the type of dependent variable (e.g., continuous, binary, count). For self-reported frequency (n_trips_/wk) and duration (hr/wk) of walking for transportation outcomes, we limit analyses to participants that reported ≥ 1 walking for transportation trip (*n* = 124, 77% of participants) in order to improve model fit.

To examine the association between Street Smart Walk Score and TAC (n/day) we used linear regression. We first fitted a crude model to estimate the main effect of Street Smart Walk Score on TAC, with Street Smart Walk Score as the only independent variable. We then fitted a second model identical to the first but controlling for the effects of age and gender. Finally, we fitted a third model identical to the second but with all independent variables associated with TAC at *p* ≤ 0.20 in bivariate analyses. We selected independent variables for bivariate analyses based on their known associations with older adult physical activity and/or walking [18, 20, 25, 42–44]. The independent variables spanned perceived built environment, neighbourhood social environment, physical, psychosocial and sociodemographic domains (described in measures).

We followed the same procedure (above) for the other continuous dependent variables [steps (n/day), light intensity physical activity (min/day), MVPA (min/day), and duration of walking for transportation (hr/wk)]. We applied a log transformation for TAC and MVPA as residuals were highly skewed. For these models we present exponentiated regression coefficients to interpret them in the original unit of measurement (n/day and min/day). These exponentiated coefficients are interpreted as fold-change in the dependent variable.

Using logistic regression and a truncated Poisson regression model, we examined the association between Street Smart Walk Score and: i) odds of any (compared with none) walking for transportation, and ii) frequency of walking for transportation (n_trip_/wk), respectively. These analyses followed the same procedure as for our continuous dependent variables. We report truncated Poisson model coefficients and associated confidence intervals transformed to incidence-rate ratios (IRRs), calculated as e^βi^.

For each outcome, we used Akaike’s information criterion to guide selection between crude and adjusted models. We assessed the adequacy of the fitted normal linear regression models with residual plots. We assessed the adequacy of the fitted logistic regression models by plots of observed versus estimated probabilities grouped into deciles of estimated probability and with the Hosmer-Lemeshow goodness of fit test. We assessed the adequacy of the fitted truncated Poisson regression models with the likelihood ratio test and comparison of standard errors and point estimates between truncated Poisson models fitted with robust standard errors and Poisson models, respectively. For each of the fully adjusted models, we calculated variance inflation factors; a variance inflation factor > 10 was regarded as indicating serious multicollinearity that warranted changes to the model. Finally, we also investigated outliers with the dfbeta command in Stata.

We considered *p* < 0.05 to be statistically significant in multivariable analyses. We conducted all analyses using Stata version 13.0 (Stata Corp, TX).

## Results

Previously we described flow of participants into the study [15]. Briefly, we randomly sampled 2000 households from our source population of 5806 households. After exclusion of five households due to prior attempted recruitment into our pilot study, we contacted 1995 individuals (from 1995 households) for study participation. All 161 individuals that consented to participate completed an in-person measurement session. All but two participants (who declined) wore an accelerometer for 1 week to capture patterns of physical activity. One hundred and fifty eight participants returned accelerometers; 141 had ≥ 3 days of valid data. Participants that provided valid accelerometry data wore accelerometers for a mean (SD) of 7 (1) days and a mean (SD) of 784 (105) minutes/day.

We present select characteristics of participants, by gender, in Table 1. Participant characteristics did not vary between those with vs. without ≥ three valid days of wear time (data not shown). Participants’ mean age was 74 years, 65% were women, and more than 3/4 (81%) lived alone. Approximately half of participants reported a vehicle at their disposal in the 7 days prior to study participation. Participants were overweight (BMI range 25.0–29.9), had a mean of three comorbidities, and had a gait speed that was consistent with community walking ( ≥ 0.8 m/s) [43], on average.

**Table 1:** Descriptive statistics for select characteristics, by gender

Characteristic	Men		Women		Total	
	n	mean (SD)	n	mean (SD)	n	mean (SD)
SOCIODEMOGRAPHICS						
Age (yrs)	59	74.2 (6.3)	102	74.4 (6.2)	161	74.3 (6.2)
Married (%)	59		102		161	
No		81		97		91
Yes		19		3		9
Living arrangement (%)	59		102		161	
Lives alone		68		88		81
Lives with others		32		12		19
Had vehicle at disposal in last 7 days (%)	59		102		161	
No		41		50		47
Yes		59		50		53
Owns a dog (%)	59		102		161	
No		92		88		89
Yes		8		12		11
BUILT ENVIRONMENT						
Street Smart Walk Score (/100)	59	71.3 (27.7)	102	71.8 (24.0)	161	71.6 (25.3)
NEWS-A^a^ Subscale F: Aesthetics (/4)	58	2.9 (0.8)	102	3.3 (0.6)	160	3.2 (0.7)
NEWS-A^a^ Subscale G: Traffic hazards (/4)	57	2.4 0.6)	98	2.4 (0.6)	155	2.4 (0.6)
NEWS-A^a^ Subscale H: Crime (/4)	56	3.3 (0.7)	96	3.3 (0.7)	152	3.3 (0.7)
PHYSICAL						
Body mass index (kg/m^2^)	59	26.9 (4.6)	102	27.0 (5.7)	161	27.0 (5.3)
Number of comorbidities^b^	57	2.8 (2.0)	101	3.0 (2.2)	158	2.9 (2.1)
Gait speed (m/s)^c^	59	1.0 (0.2)	102	1.0 (0.3)	161	1.0 (0.3)
PSYCHOSOCIAL						
Likes to walk outside… (%)	59		102		161	
Less than very much (1-4 on a 5-point scale)		44		25		31
Very much (5 on a 5-point scale)		66		75		69
Ambulatory self-confidence questionnaire (/10)	59	8.6 (1.4)	102	8.2 (1.8)	161	8.4 (1.7)
SOCIAL ENVIRONMENT						
Neighbourhood social cohesion and trust^d^ (/5)	56	3.3 (0.8)	101	3.5 (0.7)	157	3.4 (0.7)
Neighbourhood physical and social disorder^e^ (/4)	57	3.4 (0.6)	102	3.5 (0.4)	159	3.5 (0.5)

^a^NEWS-A = Neighbourhood Environment Walkability Scale – abbreviated; some scales reverse coded so that higher score indicates better walkability

^b^Total number; measured with the Functional Comorbidity Index

^c^Assessed as part of the 4-m walk (usual pace) component of the Short Physical Performance Battery

^d^5-item measure of social cohesion and trust

^e^5-item measure of neighbourhood physical and social disorder; reverse coded so that higher score indicates better walkability (less disorder)

Table 2 describes participants’ physical activity and walking for transportation. Participants engaged in approximately 240 min of physical activity/day (on average), of which 220 min was light and 20 min was moderate-to-vigorous intensity. Of note, participants obtained the vast majority of their MVPA through moderate intensity physical activity; on average, they spent less than 1 min/day in vigorous physical activity (data not shown). One hundred and twenty-four participants (77%) reported any walking for transportation in the assessment week.

**Table 2:** Physical activity and walking for transportation outcomes, by gender

Outcome	Men		Women		Total	
	n	mean (SD)	n	mean (SD)	n	mean (SD)
PHYSICAL ACTIVITY^a^	49		92		141	
TAC^c^ (n/day)		175889.5 (97991.6)		170473.3 (103190.6)		172355.5 (101095.7)
Steps (n/day)		5113 (2572)		5175 (3165)		5153 (2963)
Light physical activity (min/day)		193.1 (66.8)		234.1 (79.9)		219.8 (77.9)
MVPA^b^ (min/day)		23.5 (20.5)		17.8 (20.4)		19.8 (20.6)
WALKING FOR TRANSPORTATION^d^	59		65		124	
Frequency (n_trips_/wk)		4.4 (2.2)		4.0 (2.0)		4.2 (2.1)
Duration (hr/wk)		4.1 (3.0)		3.4 (2.6)		3.7 (2.8)

^a^As measured by accelerometry (ActiGraph GT3X+, 60 s epochs), based on ≥ 3 days with ≥ 480 min/day valid weartime

^b^MVPA = moderate-to-vigorous physical activity

^c^TAC = total activity counts

^d^As measured by the Community Healthy Activities Model Program for Seniors survey; only includes participants that reported making ≥ 1 walking for transportation trip (*n* = 124)

Tables 3 and 4 display crude and adjusted linear regression analyses for physical activity volume (TAC and steps) and intensity (light physical activity and MVPA). Street Smart Walk Score was not associated with any of these physical activity outcomes in crude or adjusted models. Among covariates, BMI was associated with all four outcomes in fully adjusted models. Each unit increase in BMI was associated with a 3% (95% CI = -4, -1) decrease in TAC, 162 (95% CI = -245, -79) fewer steps, 2.97 (95% CI = -5.38, -0.57) minutes less of light physical activity, and a 7% (95% CI = -11, -3) decrease in MVPA. Age and self-reported walking enjoyment (very much like to walk) were also associated with all physical activity outcomes except light physical activity in fully adjusted models. Each 10-year increase in age was associated with a 19% (95% CI = -30, -5) decrease in TAC, 903 (95% CI = -1642, -164) fewer steps, and a 34% (95% CI = -53, -7) decrease in MVPA. Very much liking to walk (vs. less than very much liking to walk) was associated with a 32% (95% CI = 7, 63) increase in TAC, taking 1342 (95% CI = 337, 2346) more steps, and a 100% (95% CI = 25, 221) increase in MVPA. Women engaged in 34.09 (95% CI = 5.67, 62.50) more minutes of light physical activity and 47% (95% CI = -66, -16) less MVPA compared with men (fully adjusted models). Finally, MVPA increased by 193% (95% CI = 25, 221) for each unit increase in gait speed (fully adjusted models).

**Table 3:** Estimates from linear regression analyses for physical activity volume [total activity counts (TAC, number/day)^a^ and steps (number/day)]

Predictor	TAC (n/day)						Steps (n/day)					
	Crude		Adjusted				Crude		Adjusted			
			Model 2^c^		Model 3^d^				Model 4^c^		Model 5^e^	
	(*n* = 141)^b^		(n = 141)^b ^fit on a line		(*n* = 131)		(*n* = 141)^b^		(*n* = 141)		(*n* = 138)	
	β (95% CI)	*P*	β (95% CI)	*P*	β (95% CI)	*P*	β (95% CI)	*P*	β (95% CI)	*P*	β (95% CI)	*P*
Street Smart Walk Score (10-point change)	1.01 (0.97, 1.05)	0.546	1.02 (0.99, 1.07)	0.221	1.00 (0.96, 1.04)	0.991	51 (−148, 250)	0.612	102 (−93, 297)	0.304	−23 (−207, 160)	0.801
Women	0.98 (0.8, 1.21)	0.876	0.98 (0.8, 1.19)	0.816	0.90 (0.74, 1.10)	0.308	61 (−978, 1101)	0.907	31 (−975, 1038)	0.951	−347 (−1309, 616)	0.478
Age (10-year change)	*0.74* (0.63, 0.87)*	<0.001	*0.73* (0.62, 0.85)*	<0.001	*0.81* (0.70, 0.95)*	0.011	−*1298* (−2091,−505)*	0.002	−*1362* (−2168,−557)*	0.001	−*903* (−1642,−164)*	0.017
Vehicle available	0.86 (0.71, 1.06)	0.156	-	-	0.94 (0.79, 1.12)	0.495	−695 (−1687, 297)	0.168	-	-	−293 (−1150, 565)	0.500
Aesthetics^f^	*1.23* (1.07, 1.42)*	0.004	-	-	1.08 (0.94, 1.23)	0.272	*879* (178, 1580)*	0.014	-	-	233 (−415, 881)	0.478
Crime^g^	1.11 (0.96, 1.28)	0.173	-	-	1.07 (0.95, 1.22)	0.272	-	-	-	-	-	-
Body mass index (kg/m^2^)	*0.96* (0.94, 0.97)*	<0.001	-	-	*0.97* (0.96, 0.99)*	0.004	−*234* (−316,−152)*	<0.001	-	-	−*162* (−245,−79)*	<0.001
Comorbidities^h^	*0.92* (0.87, 0.96)*	<0.001	-	-	0.97 (0.92, 1.02)	0.180	−*399* (−625,−173)*	0.001	-	-	−120 (−336, 97)	0.276
Gait speed (m/s)^i^	*2.79* (1.94, 4.02)*	<0.001	-	-	1.40 (0.90, 2.18)	0.140	*4816* (2997, 6635)*	<0.001	-	-	1738 (−397, 3873)	0.110
Very much like to walk^j^	*1.67* (1.37, 2.04)*	<0.001	-	-	*1.32* (1.07, 1.63)*	0.010	*2495* (1519, 3471)*	<0.001	-	-	*1342* (337, 2346)*	0.009
Ambulatory Confidence^k^	*1.13* (1.06, 1.19)*	<0.001	-	-	1.01 (0.95, 1.09)	0.690	*546* (259, 834)*	<0.001	-	-	98 (223, 418)	0.547

^a^TAC (number/day) presented as exponentiated regression coefficients

^b^n_vehicle available_ = 140; n_aesthetics_ = 140; n_traffic hazards_ = 135; n_comorbidities_ = 139

^c^Adjusted for Street Smart Walk Score, gender, age

^d^Adjusted for all predictor variables listed in this table

^e^Adjusted for all predictor variables listed in this table with the exception of crime, since crime was not associated with steps (n/day) at *p* ≤ 0.2 in bivariate analyses

^f^ Neighbourhood Environment Walkability Scale—abbreviated (NEWS-A) Subscale F: Aesthetics (four-point scale); reverse coded so that higher score indicates better walkability

^g^NEWS-A Subscale H: Crime (four-point scale); reverse coded so that higher score indicates better walkability

^h^Total number; measured with the Functional Comorbidity Index

^i^Assessed as part of the 4-m walk (usual pace) component of the Short Physical Performance Battery

^j^Very much like to walk (5 on a 5-point scale) vs. less than very much liking to walk (1–4 on a 5-point scale)

^k^Assessed by the Ambulatory Self-Confidence Questionnaire

*p < 0.05

**Table 4:** Estimates from linear regression analyses for physical activity intensity [light physical activity (minutes/day) and moderate-to-vigorous physical activity (MVPA, minutes/day)^a^]

	Light physical activity (min/day)						MVPA (min/day)					
Predictor	Crude		Adjusted				Crude		Adjusted			
			Model 2^c^		Model 3^d^				Model 4^c^		Model 5^e^	
	(*n* = 141)^b^		(*n* = 141)		(*n* = 134)		(*n* = 141)^b^		(*n* = 141)		(*n* = 138)	
	β (95% CI)	*P*	β (95% CI)	*P*	β (95% CI)	*P*	β (95% CI)	*P*	β (95% CI)	*P*	β (95% CI)	*P*
Street Smart Walk Score (10-point change)	−3.48 (−8.69, 1.73)	0.189	−2.73 (−7.80, 2.33)	0.288	−5.22 (−10.83, 0.39)	0.068	1.04 (0.95, 1.15)	0.395	1.07 (0.97, 1.18)	0.164	1.00 (0.92, 1.09)	0.950
Women	*40.93* (14.48, 67.38)*	0.003	*40.55* (14.44, 66.66)*	0.003	*34.09* (5.67, 62. 50)*	0.019	0.70 (0.42, 1.17)	0.175	0.69 (0.42, 1.13)	0.144	*0.53* (0.34, 0.84)*	0.007
Age (10-year change)	*−22.97* (−44.24,−1.71)*	0.034	−20.72 (−41.61, 0.18)	0.052	−6.20 (−29.20, 16.81)	0.595	*0.54* (0.36, 0.79)*	0.002	*0.51* (0.34, 0.76)*	0.001	*0.66* (0.47, 0.93)*	0.019
Vehicle available	-	-	-	-	-	-	*0.61* (0.37, 0.99)*	0.045	-	-	0.71 (0.48, 1.06)	0.097
Aesthetics^f^	*19.58* (1.00, 38.15)*	0.039	-	-	6.26 (−13.03, 25.55)	0.522	*1.56* (1.10, 2.20)*	0.012	-	-	1.18 (0.87, 1.60)	0.275
Traffic hazards^g^	17.82 (−5.43, 41.07)	0.132	-	-	11.33 (−10.79, 33.45)	0.313	-		-	-	-	-
Body mass index (kg/m^2^)	*−3.59* (−5.90,−1.28)*	0.003	-	-	*−2.97* (−5.38,−0.57)*	0.016	*0.89* (0.85, 0.93)*	<0.001	-	-	*0.93* (0.89, 0.97)*	<0.001
Comorbidities^h^	*−6.44* (−12.55,−0.34)*	0.039	-	-	−1.97 (−8.46, 4.52)	0.549	*0.80* (0.71, 0.89)*	<0.001	-	-	0.92 (0.83, 1.02)	0.108
Gait speed (m/s)^i^	*101.82* (52.37, 151.27)*	<0.001	-	-	59.43 (−2.77, 121.63)	0.061	*11.97* (4.91, 29.17)*	<0.001	-	-	*2.93* (1.08, 7.98)*	0.035
Very much like to walk^j^	*36.86* (9.63, 64.08)*	0.008	-	-	10.59 (−19.24, 40.43)	0.483	*3.57* (2.21, 5.76)*	<0.001	-	-	*2.00* (1.25, 3.21)*	0.004
Ambulatory confidence^k^	6.83 (−1.02, 14.69)	0.088	-	-	0.78 (−8.74, 10.31)	0.871	*1.33* (1.16, 1.53)*	<0.001	-	-	1.02 (0.87, 1.18)	0.844

^a^MVPA (min/day) presented as exponentiated regression coefficients

^b^n_vehicle available_ = 140; n_aesthetics_ = 140; n_traffic hazards_ = 135; n_comorbidities_ = 139

^c^adjusted for Street Smart Walk Score, gender, and age

^d^adjusted for all variables listed in this table with the exception of vehicle availability, since vehicle availability was not associated with time spent in light physical activity (min/day) at *p* ≤ 0.2 in bivariate analyses

^e^adjusted for all variables listed in this table with the exception of traffic, since traffic was not associated with time spent in MVPA (min/day) at *p* ≤ 0.2 in bivariate analyses

^f^NEWS-A (Neighbourhood Environment Walkability Scale—abbreviated) Subscale F: Aesthetics (four-point scale)

^g^NEWS-A Subscale G: Traffic hazards (four-point scale); reverse coded so that higher score indicates better walkability

^h^Total number; measured with the Functional Comorbidity Index

^i^Assessed as part of the 4-m walk (usual pace) component of the Short Physical Performance Battery

^j^Very much like to walk (5 on a 5-point scale) vs. less than very much liking to walk (1–4 on a 5-point scale)

^k^Assessed by the Ambulatory Self-Confidence Questionnaire

*p < 0.05

Table 5 highlights the crude and adjusted results of logistic regression models for any walking for transportation. In the fully adjusted model, the odds of any walking for transportation was 1.45 (95% CI = 1.18, 1.78) times greater for each 10-point increase in Street Smart Walk Score. Further, in this model, the odds of any walking for transportation were 14.29 (95% CI = 3.33, 50.00) times higher among participants who did not have a vehicle available and 5.60 (95% CI = 1.68, 18.65) higher for those who very much liked to walk. No other variables were associated with any walking for transportation in fully adjusted models.

**Table 5:** Estimates from logistic regression analyses for making any walking for transportation trip/wk (vs. none)

	Crude				Adjusted	
			Model 2^b^		Model 3^c^	
	(*n* = 161)^a^ OR (95% CI)	*P*	(*n* = 161) OR (95% CI)	*P*	(*n* = 151) OR (95% CI)	*P*
Street Smart Walk Score (10-point change)	*1.37* (1.18, 1.59)*	<0.001	*1.37* (1.18, 1.60)*	<0.001	*1.45* (1.18, 1.78)*	<0.001
Women	1.24 (0.58, 2.63)	0.576	1.21 (0.54, 2.72)	0.846	0.97 (0.32, 2.97)	0.571
Age (10-year change)	1.09 (0.60, 1.98)	0.772	0.94 (0.49, 1.80)	0.648	1.29 (0.54, 3.07)	0.963
Vehicle available	*0.09* (0.03, 0.28)*	<0.001	-	-	*0.07* (0.02, 0.30)*	<0.001
Aesthetics^d^	1.46 (0.89, 2.40)	0.136	-	-	1.15 (0.50, 2.61)	0.746
Comorbidities^e^	0.85 (0.72, 1.01)	0.068	-	-	0.88 (0.68, 1.14)	0.335
Gait speed (m/s)^f^	3.61 (0.79, 16.51)	0.098	-	-	1.71 (0.10, 29.34)	0.713
Very much like to walk^g^	*4.30* (1.99, 9.30)*	<0.001	-	-	*5.60* (1.68, 18.65)*	0.005
Ambulatory confidence^h^	1.17 (0.95, 1.45)	0.134	-	-	0.97 (0.68, 1.40)	0.885
Social cohesion^i^	0.60 (0.34, 1.03)	0.065	-	-	0.49 (0.23, 1.05)	0.067
Disorder^j^	*0.36* (0.14, 0.89)*	0.028	-	-	0.67 (0.16, 2.73)	0.572

^a^n_vehicle available_ = 159; n_aesthetics_ = 160; n_comorbidities_ = 158; n_social cohesion_ = 157; n_disorder_ = 159

^b^adjusted for Street Smart Walk Score, gender, and age

^c^adjusted for all predictor variables listed in this table

^d^Neighbourhood Environment Walkability Scale—abbreviated (NEWS-A) Subscale F: Aesthetics (four-point scale); reverse coded so that higher score indicates better walkability

^e^Total number; measured with the Functional Comorbidity Index

^f^Assessed as part of the 4-m walk (usual pace) component of the Short Physical Performance Battery

^g^Very much like to walk (5 on a 5-point scale) vs. less than very much liking to walk (1–4 on a 5-point scale)

^h^Assessed by the Ambulatory Self-Confidence Questionnaire

^i^5-item measure of social cohesion and trust

^j^5-item measure of neighbourhood physical and social disorder; reverse coded so that higher score indicates better walkability (less disorder)

*p < 0.05

Table 6 shows the crude and adjusted results of truncated Poisson regression models and linear regression models for frequency (n_trips_/wk) and duration (hr/wk) of walking for transportation, respectively. These models include only participants who reported any walking for transportation (*n* = 124). Although Street Smart Walk Score was associated with *frequency* of walking for transportation in the crude model (IRR = 1.06, 95% CI = 1.01, 1.10) and the model adjusted for age and gender (IRR = 1.06, 95% CI = 1.01, 1.11), it was no longer significant in the fully adjusted model (IRR = 1.03, 95% CI = 0.98, 1.08). Street Smart Walk Score was not associated with *duration* of walking for transportation in any model. In fully adjusted models, very much like to walk (vs. less than very much like to walk) increased the incidence rate of frequency of walking for transportation by 1.52 (95% CI = 1.15, 2.01) and duration of walking for transportation by 1.44 (95% CI = 0.15, 2.73). Further, each unit increase in BMI was associated with a 0.11 (95% CI = -0.21, -0.01) decrease in duration of walking for transportation.

**Table 6:** ^a^Estimates from regression analyses for frequency (n_trips_/wk)^b^ and duration (hr/wk)^c^ of walking for transportation

			Frequency (n_trips_/wk)						Duration (hr/wk)			
	Crude				Adjusted		Crude				Adjusted	
			Model 2^e^		Model 3^f^				Model 4^e^		Model 5^h^	
Predictor	(*n* = 124)^d^ IRR (95% CI)	*P*	(*n* =124) IRR (95% CI)	*P*	(*n* =121) IRR (95% CI)	*P*	(*n* = 124)^g^ β (95% CI)	*P*	(*n* = 124) β (95% CI)	*P*	(*n* = 112) β (95% CI)	*P*
Street Smart Walk Score (10-point change)	*1.06* (1.01, 1.10)*	0.013	*1.06* (1.01, 1.11)*	0.011	1.03 (0.98, 1.08)	0.206	−0.01 (−0.23, 0.21)	0.920	−0.01 (−0.23, 0.21)	0.956	−0.01 (−0.27, 0.25)	0.935
Women	0.89 (0.74, 1.07)	0.228	0.89 (0.74, 1.08)	0.237	0.87 (0.72, 1.06)	0.181	−0.69 (−1.71, 0.34)	0.187	−0.68 (−1.71, 0.35)	0.196	−0.81 (−1.89, 0.28)	0.145
Age (10-year change)	0.94 (0.81, 1.09)	0.437	0.93 (0.80, 1.08)	0.354	1.04 (0.89, 1.23)	0.608	−0.21 (−1.02, 0.60)	0.613	−0.19 (−1.00, 0.62)	0.643	0.12 (−0.76, 1.00)	0.786
Vehicle available	-	-	-	-	-	-	−0.98 (−1.97, 0.01)	0.053	-	-	−0.68 (−1.78, 0.41)	0.217
Crime^i^	-	-	-	-	-	-	*−0.79* (−1.52,−0.06)*	0.034	-	-	−0.76 (−1.62, 0.09)	0.079
Body mass index (kg/m^2^)	*0.98* (0.96, 1.00)*	0.041	-	-	0.99 (0.97, 1.01)	0.420	−0.09 (−0.19, 0.00)	0.058	-	-	*−0.11* (−0.21,−0.01)*	0.046
Comorbidities^j^	0.96 (0.92, 1.01)	0.097	-	-	1.00 (0.95, 1.06)	0.989	-	-	-	-	-	-
Very much like to walk^k^	*1.66* (1.28, 2.14)*	<0.001	-	-	*1.52* (1.15, 2.01)*	0.004	*1.29* (0.15, 2.44)*	0.027	-	-	*1.44* (0.15, 2.73)*	0.029
Ambulatory confidence^l^	*1.09* (1.02, 1.16)*	0.010	-	-	1.05 (0.98, 1.13)	0.184	-	-	-	-	-	-
Social Cohesion^m^	-	-	-	-	-	-	*−0.81* (−1.55,−0.06)*	0.034	-	-	−0.65 (−1.45, 0.15)	0.109
Disorder^n^	*0.80* (0.68, 0.95)*	0.009	-	-	1.19 (0.99, 1.43)	0.057	−0.71 (−1.68, 0.25)	0.144	-	-	0.23 (−0.96, 1.43)	0.699

^a^These analyses only include participants (*n* = 124) that self-reported ≥ 1 walking for transportation trip [as measured by the Community Healthy Activities Model Program for Seniors (CHAMPS) survey]

^b^Analysed using truncated poisson regression models. Data are presented as incident rate ratios (IRRs)

^c^Analysed using linear regression models

^d^n_crime_ = 117; n_comorbidities_ = 121; n_disorder_ = 123

^e^adjusted for Street Smart Walk Score, gender, and age

^f^adjusted for all predictor variables listed in this table with the exception of vehicle availability, crime, and social cohesion, since these three variables were not associated with frequency of walking for transportation (n_trips_/wk) at *p* ≤ 0.2 in bivariate analyses

^g^n_vehicle available_ = 123; n_crime_ = 117; n_social cohesion_ = 120; n_disorder_ = 123

^h^adjusted for all predictor variables listed in this table with the exception of comorbidities and ambulatory confidence, since these two variables were not associated with duration of walking for transportation (hr/wk) at *p* ≤ 0.2 in bivariate analyses

^i^Neighbourhood Environment Walkability Scale—abbreviated (NEWS-A) Subscale H: Crime (four-point scale); reverse coded so that higher score indicates better walkability

^j^Total number; measured with the Functional Comorbidity Index

^k^Very much like to walk (5 on a 5-point scale) vs. less than very much liking to walk (1–4 on a 5-point scale)

^l^Assessed by the Ambulatory Self-Confidence Questionnaire

^m^5-item measure of social cohesion and trust

^n^5-item measure of neighbourhood physical and social disorder; reverse coded so that higher score indicates better walkability (less disorder)

*p < 0.05

## Discussion

We noted that a walkable neighbourhood (as measured by Street Smart Walk Score) was associated with significantly higher odds of engaging in any self-reported walking for transportation, but not with the volume or intensity of physical activity (as measured by accelerometry). Further, among those who walked for transportation, there was no association between neighbourhood walkability and frequency or duration of walking for transportation. As we found no published studies of older adults living on low income, we compare our findings to studies conducted in a general older adult population; that is, community-dwelling older adults who were not recruited in reference to a specific characteristic (like, disease status, SES, etc.). Importantly, we note the tremendous diversity of outcomes reported and instruments used to measure physical activity and the built environment.

Seven studies used objective measures of the built environment and assessed physical activity objectively (by accelerometry) in older adults. Five of these [45–50] used a composite index for neighbourhood walkability and all were observational trials. Buman and colleagues reported the importance of light intensity physical activity to the physical health and psychosocial well-being of older adults [8]. Despite this, the current literature (and recommended guidelines) tend to focus on cardiovascular benefits of physical activity and thus the association between objectively measured features of the built environment and time spent in MVPA; findings were mixed [45–47, 49, 50]. Only one study reported older adults’ light intensity physical activity, divided into low-light and high-light physical activity intensity [50]. They reported a significant negative association between low-light physical activity and objectively measured walkability and no association between walkability and high-light physical activity [50]. Other studies investigated the association between total physical activity volume (by accelerometry) and objectively measured walkability [47, 48], also with mixed results. Collectively, these findings speak to a complex association between the built environment and older adults’ time spent in physical activity. Given the many factors that contribute to older adults’ physical activity, finding no clear association between the built environment and physical activity is not uncommon.

There are a few potential explanations for the lack of significant associations between neighbourhood walkability and physical activity volume and intensity. First, an individual’s activity is a product of the dynamic interplay between characteristics of the individual and features of the environment [21]. Thus the association between the built environment and older adult physical activity may be moderated by person-level variables. For example, Ding and colleagues found that time spent in MVPA was significantly associated with walkability among drivers, but not non-drivers [47, 50]. Van Holle and colleagues found that time spent in MVPA was only associated with walkability in high walkability/low neighbourhood income areas [47, 50]. Second, physical activity is a broad construct that encompasses four domains—leisure time physical activity, occupational physical activity, household physical activity and transportation-related physical activity [42]. It could be that older adults who live in low walkable neighbourhoods supplement their physical activity with activities that take place outside of an undesirable neighbourhood built environment. Van Holle and colleagues suggested that older adults who live in less walkable neighbourhoods may spend more time engaged in indoor activities, such as housework [50]. Finally, older adults who live in more walkable neighbourhoods may make shorter trips to nearby, accessible destinations. Therefore, they accrue less physical activity in their daily travel than counterparts who live in less walkable neighbourhoods and thus travel further to reach amenities. This is consistent with a previous study from our group that demonstrated neighbourhood walkability was associated with smaller activity spaces [51].

Importantly, living in a more walkable neighbourhood was associated with greater odds of older adults doing any walking for transportation. Thus, low walkable neighbourhoods may act as a barrier to older adults’ decision to walk for transportation. Alternatively, more walkable neighbourhoods may enable older adults to integrate active transportation into their daily life routines. However, we did not find a dose-response among those who made transportation-related walking trips. Previous studies reported positive associations between older adults’ self-reported time spent walking for transportation and objectively measured walkability [14, 45, 46, 49, 50]. Unique to our study, we included only participants living on low income who made ≥ one walking for transportation trip. Previously, we reported an association between objectively measured walkability and frequency of walking trips [15]. This study extends our previous study as we use a different instrument (self-report physical activity questionnaire vs. travel diaries) and focus our analyses on participants who engaged in any walking for transportation.

The interaction between a person and their environment is dynamic and together they influence [walking] behavior [21]. Our findings at the person-level underscore this dynamism. More specifically, sociodemographic, physical function and attitudinal factors are established predictors of older adult physical activity [42–44]. All surfaced as significant predictors of physical activity and/or walking for transportation in our regression models. As per other reports [52, 53], our results were gender-specific in that men spent more time engaged in MVPA and less time in light intensity physical activity compared with women. Age was inversely associated with all levels of physical activity (by accelerometry) except that of light intensity. This speaks to an older adult’s ability to sustain light activities over time whereas the ability to engage in more intense activities may decline. Body mass index was inversely associated with all four physical activity outcomes and duration of walking for transportation. Gait speed was positively associated with MVPA. This speaks to the close link between physical capacity and mobility and one’s engagement in higher intensity physical activities. Finally, an attitudinal factor (how much participants liked to walk) was associated with all outcomes except for light physical activity.

We note that our study has some strengths. These include: i) our focus on an understudied and potentially “at risk” population (older adults living on low income), ii) reporting outcomes across a spectrum of physical activity -- the importance of light activity and physical activity volume are, in our view, currently understudied, iii) the selection of independent variables for our models based on a theoretical framework [23], iv) a robust sampling frame that included stratification across deciles of neighbourhood walkability to ensure variability in urban form, v) objective measures of physical activity volume and intensity in real-time (by accelerometry), vi) complementing objective measures of physical activity with self-reported data in order to capture domain-specific physical activity (walking for transportation), as this may be more closely associated with the built environment [54], and vii) including perceived measures of the built environment to complement objective measures.

We acknowledge that our study also had a number of limitations. Our recruitment rate was only 8%. This relatively low response rate likely reflects our focus upon a low SES population, a group that tends to not participate in research [55]. Self-selection bias may exist if active, healthy participants were more likely to participate. That said, key determinants of physical activity (e.g., age and gender) in study participants were similar to those of the source population (SAFER recipients). Finally, in order to improve model fit, we limited analyses for self-reported frequency and duration of walking trip to participants that reported making ≥ 1 walking for transportation trip. This limits the generalizability of the findings of these two analyses to individuals who leave the home.

## Conclusions

A more walkable neighbourhood appears to be a worthwhile investment as it encourages older adults living on low income to walk for transportation. However, factors beyond the built environment alone appear to influence duration and frequency of these trips and physical activity in general. This is best investigated in future, through built environment studies with larger, diverse samples of older adults living on low income that might also provide the opportunity to investigate moderating relations.

In our view, future studies that consider or incorporate the following would be of great benefit: i) how the ratio of indoor and outdoor physical activity differs between residents of highly walkable and low walkable neighbourhoods; ii) characterizing older adult movement and physical activity using a combination of accelerometry and global positioning systems to investigate walking trip lengths, as well as the association between walking for transportation and physical activity, across a range of walkability; and iii) investigating the association between person and environment-level variables on light physical activity as measured by accelerometry. We live in a time when the health and mobility of an aging demographic will dictate the demands placed upon municipal and provincial governments. Therefore it seems crucial to identify aspects of the built environment that promote healthy behaviours, like physical activity, across a broad (economic and health) spectrum of older adults as a means to guide these decision makers.

## Abbreviations

BMI: Body mass index
CHAMPS: Community Healthy Activities Model Program for Seniors survey
CI: Confidence interval
IRR: Incident rate ratio
MVPA: Moderate-to-vigorous physical activity
NEWS-A: Neighbourhood Environment Walkability Scale—abbreviated
SAFER: Shelter Aid for Elderly Renters
SD: Standard deviation
SES: Socioeconomic status
TAC: Total activity counts
